# Towards the Extraction of Radioactive Cesium-137 from Water via Graphene/CNT and Nanostructured Prussian Blue Hybrid Nanocomposites: A Review

**DOI:** 10.3390/nano9050682

**Published:** 2019-05-02

**Authors:** Protima Rauwel, Erwan Rauwel

**Affiliations:** Institute of Technology, Estonian University of Life Sciences, Kreutzwaldi 56/1, 51014 Tartu, Estonia; erwan.rauwel@emu.ee

**Keywords:** carbon nanotubes, graphene, Prussian blue, 137-Cesium, water remediation, magnetic extraction, ^137^Cs^+^ selectivity, radioactive contamination

## Abstract

Cesium is a radioactive fission product generated in nuclear power plants and is disposed of as liquid waste. The recent catastrophe at the Fukushima Daiichi nuclear plant in Japan has increased the ^137^Cs and ^134^Cs concentrations in air, soil and water to lethal levels. ^137^Cs has a half-life of 30.4 years, while the half-life of ^134^Cs is around two years, therefore the formers’ detrimental effects linger for a longer period. In addition, cesium is easily transported through water bodies making water contamination an urgent issue to address. Presently, efficient water remediation methods towards the extraction of ^137^Cs are being studied. Prussian blue (PB) and its analogs have shown very high efficiencies in the capture of ^137^Cs^+^ ions. In addition, combining them with magnetic nanoparticles such as Fe_3_O_4_ allows their recovery via magnetic extraction once exhausted. Graphene and carbon nanotubes (CNT) are the new generation carbon allotropes that possess high specific surface areas. Moreover, the possibility to functionalize them with organic or inorganic materials opens new avenues in water treatment. The combination of PB-CNT/Graphene has shown enhanced ^137^Cs^+^ extraction and their possible applications as membranes can be envisaged. This review will survey these nanocomposites, their efficiency in ^137^Cs^+^ extraction, their possible toxicity, and prospects in large-scale water remediation and succinctly survey other new developments in ^137^Cs^+^ extraction.

## 1. Introduction

Cesium-137 is a radioactive element with a half-life of 30.4 years emitting both beta and gamma radiations. Under normal circumstances, its release is an outcome of radioactive testing of fission reactions, whereby making it the most abundant radioactive atmospheric pollutant capable of entailing health hazards. The ^137^Cs isotope is produced when uranium and plutonium undergo fission after having absorbed neutrons in a nuclear reactor and is detectable and measurable by gamma counting. It decays into ^137^Ba with a half-life of 2.6 min. Small amounts of ^137^Cs^+^ are released on a regular basis in spent fuel ponds through cracks in the fuel rod that reach the coolant and fuel reprocessing waters, which are all subsequently discharged into the sea as effluents [1]. Today, in view of the Fukushima-Daiichi catastrophe, the extraction of radioactive cesium from soil and water is a necessity and calls for the development of new technologies. The projected radiation effects of ^137^Cs will remain at their maximum for the next 100 years, seconded by isotopes of strontium and plutonium. Other sources of ^137^Cs contamination can be attributed to the Chernobyl accident in 1986; [2] it took 10 years for the levels to drop but their effects are still being perceived even today [3]. In general, ^137^Cs is not very mobile and tends to accumulate on the soil surface and is subsequently absorbed by plants and also captured by fungi [4]. A soil cleanup of 40,000 km^2^ reduced the radioactive contamination to 1/10th of the original value in Chernobyl.

^137^Cs is the primary cause of water contamination since the recent Fukushima Daiichi catastrophe posing more far-reaching threats than via soil contamination [5], which tends to be more localized [6]. ^137^Cs decontamination has now become a priority recommended by the International Atomic Energy Agency [7]. Mobility of ^137^Cs^+^ through moving water bodies displaces the contamination to other areas, thus widening the ‘contamination reduction’ zone. Therefore, aquatic life and fish-farming products are also contaminated, which are thereafter consumed by humans. Additionally, ^137^Cs^+^ behaves very similarly to K^+^ and Na^+^, thus facilitating its digestion and assimilation in living organisms [8]. Consumption of contaminated game and fish would introduce ^137^Cs in the human body, which would then emit harmful radiations directly targeting the cell nucleus. Moreover, in aqueous media ^137^Cs^+^ ions tend to be rather robust and unaffected by changes in pH and redox conditions, whereby making them a menace [9].

Several techniques were developed for the extraction of ^137^Cs^+^ from water: reverse osmosis [10], coagulation-sedimentation [11], ion-exchange [12], nanofiltration [13], electro-dyalysis [14] and more recently, fibrous zeolite-polymer composites [15]. On the other hand, adsorption is a highly efficient and cost-effective process with very high ion selectivity provided that the right sorbents are used [16]. Various adsorbents: inorganic adsorbents, polymer-inorganic adsorbents [7] and bioadsorbents [17], have shown efficiency in ^137^Cs^+^ extraction. Bioadsorbents however, have several disadvantages such as low sorption efficiency, especially in the presence of other salts in the aqueous medium viz., Na^+^ and K^+^. They also suffer from degradation in extreme conditions (high temperature and low pH). On the other hand, inorganic sorbents have shown large-scale applicability with high cation sorption capacities (clays viz., zeolites, bentonite and coal). However, activation of sorption sites is necessary through a surface functionalization treatment, which conversely, increases their production cost. For nanozeolites, the Q_max_ or maximum adsorption capacity values could reach up to 69 mg/g, but on the other hand, it is difficult to recover the exhausted material. Moreover, the *K*_d_ value, which is the ratio of the equilibrium adsorption of the sorbent to the equilibrium concentration of the solute, is low, implying an ineffectiveness in high solute concentrations [18]. Finally yet importantly, they do not withstand harsh aqueous environments. Synthetic polymers combined with inorganic materials seem to be more robust with a better sorption capacity than inorganic sorbents; they also show stability in harsh environments and have been already applied on a large scale in Fukushima [7].

Among the various methods available for the extraction of ^137^Cs^+^ from aqueous solutions, hybrid materials manifest enormous potential in selectively targeting and extracting ^137^Cs^+^ ions [19]. Inorganic ligands such as macrocyclic o-benzo-p-xylyl-22-crown-6-ether (OBPX22C6) ligand bonded to the hydroxyl groups of the mesoporous silica, exhibited a yield of 60% attributed to the Cs-π interaction of the OBPX22C6 benzene. Zeolite-Poly(ethersulfone) composite fiber having 30 wt % loading showed excellent properties for the decontamination of radioactive ^137^Cs^+^ [7]. The decontamination with such composites has also been demonstrated for a contamination of 823Bq/L with pH = 12. Other silicates have also been employed for their known selectivity to ^137^Cs^+^; these include crystalline silicotitanate [20,21], sodium mica [22] and sodium zirconium silicate [23]. Since they are usually in the form of very fine powders, they are therefore unsuitable for column loading. Moreover, they are difficult to separate from aqueous solutions by filtration or centrifugation therefore, reclaiming them once expended becomes problematic.

**Figure 1:**
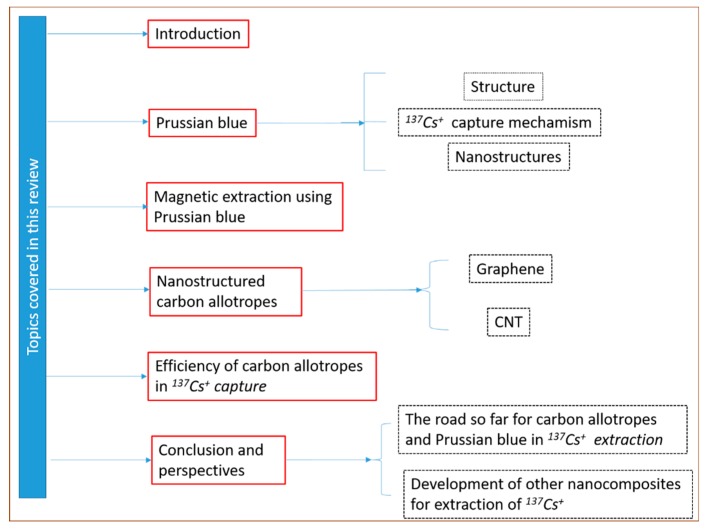
Topics covered in this review.

This review will focus on the recent developments in the extraction of ^137^Cs^+^ from water as depicted in the schematic outline of Figure 1. It will more specifically survey PB-CNT-Graphene based nanocomposite efficiencies in ^137^Cs^+^ extraction. Large-scale applicability in real case scenarios of such nanocomposites will be probed and their nanotoxicity issues will be discussed. A short summary of other new materials is also provided at the end, which opens-up new possibilities in combining these new materials with PB-CNT-graphene based nanocomposites.

## 2. Prussian Blue

### 2.1. Structure

**Figure 2:**
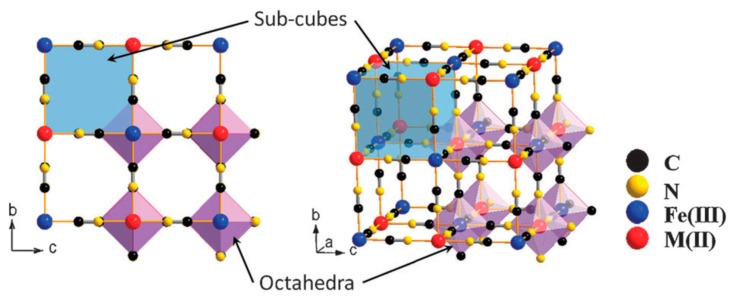
Framework of Prussian blue analogues. Adapted with permission from [27], Copyright RSC, 2012.

Prussian blue (PB) is a dark blue pigment synthesized by ferrous ferrocyanide salts with chemical formula Fe_7_(CN)_18_. It has a porous structure with the capacity to adsorb the ^137^Cs^+^ ions into its pores and store them there. It is a metal organic framework (MOF) where the inorganic vertices, which donate electrons in the structure, are linked to each other via organic compounds. The complete chemical formula is Fe^III^_4_[Fe^II^(CN)_6_]_3_·*x*H_2_O. The compound has a face-centered cubic structure (FCC) structure (Figure 2) belonging to the Fm3̅m space group with a lattice parameter of 10.166 Å. Fe exists in two oxidation states within the structure: Fe^3+^ and Fe^2+^. These ions form two different FCC lattices displaced by half a lattice parameter with respect to each other. However, the bi and tri-valent Fe are coordinated differently. Furthermore, they are linked to each other via cyanide groups (C ≡ N) i.e., C groups are linked to Fe^2+^ and N groups to Fe^3+^ with high and low spins respectively, in octahedral configurations. The Fe^2+^ and Fe^3+^ ratio of 3:4 implies that in order to obtain a charge neutrality within the structure a 25% vacancy of [Fe^+2^(CN)_6_]^4−^ molecules is necessary [24]. Coordinated water molecules occupy the resulting octahedral cavities created by such vacancies; six water molecules are linked to Fe^2+^. The other interstitial water molecules occupy the eight corners of the unit cell (¼, ¼, ¼) and are essential for the insertion of the ^137^Cs^+^ ions in the structure.

Fe^+2^ can be replaced by other transition metals with the same +2 oxidation states such as Ni, Mn, Cu and Co, coordinated exactly like Fe^+2^ in the structure and are called PB analogs. However, there are reports of Cd and Zn with slightly larger atomic radii also being incorporated into the structure owing to their +2 oxidation state [25]. The aim in including different species into the structure is to provoke a distortion of the PB lattice by producing vacancies of the high spin state molecule along with distortions in the vacant cages, in order to facilitate the capture and sequestration of the ^137^Cs^+^ [26].

### 2.2. ^137^Cs^+^ Ion Capture Mechanism in Prussian Blue

The compound is insoluble in water and the basic mechanism consists of ion exchange of ^137^Cs^+^ and H^+^ with the former occupying hydrophilic vacancies [28]. PB analogs have very different mechanisms of ion exchange or capture depending upon the anionic and alkali metal cation concentrations. Since PB and its analogs contain large amounts of interstitial and coordinated water, ^137^Cs^+^ is captured by a defect created by a [Fe^+2^(CN)_6_] vacancy, which creates a spherical cavity whose size is equivalent to the hydration radius of ^137^Cs^+^. Nevertheless, recent calculations have demonstrated that a completely dehydrated ^137^Cs^+^ ion can be incorporated into the structure with the release of a water molecule from the interstitial sites [29]. This is similar to certain clays, where on dehydrating the interlayers the ^137^Cs^+^ selectivity increases [30]. On the other hand, water soluble analogs such as metal hexacyanoferrates (HCF) consisting of a alkali metal cation with a [Fe^+2^(CN)_6_] anion, used for the extraction of ^137^Cs^+^ have shown less efficiency. In such compounds Na^+^ or K^+^ are incorporated during the synthesis of the MOF in order to render them water-soluble [31]. In addition to ^137^Cs^+^ capture mechanisms for non-soluble analogs; the water-soluble analogs mainly depend on the Na^+^ or K^+^ ion exchanges with Cs^+^. Takahashi et al., have studied the ^137^Cs^+^ uptake in KCuHCF PB analog in order to understand their lower adsorption capacity [31]. Three main mechanisms governed the ^137^Cs^+^ ion exchange according to them, with the ^137^Cs^+^-K^+^ ion exchanges being predominant, as also stipulated by other research groups. In case of low anionic vacancies, the percolation of ^137^Cs^+^ through the vacancies was prevalent. Finally, for low K^+^ incorporation in the structure, proton exchange between ^137^Cs^+^ and K^+^ ions was evidenced. Ayrault et al., report a degradation in the crystal structure of the KCuHF soluble compound after ^137^Cs^+^ adsorption which was not observed in the non-soluble counterpart [32].

### 2.3. Nanostructured Prussian Blue

**Figure 3:**
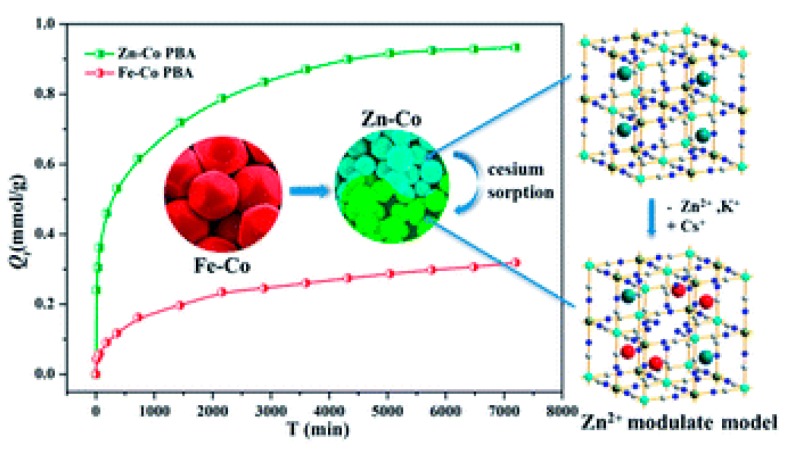
Adsorption efficiency of Fe-Co and Zn-Co prussian blue (PB) analogs as a function of time. The Zn-Co PB analog exhibits a higher Q_t_ (Q_t_ is the adsorption capacity per unit gram of the sorbent at a given time t) owing to the reduction in size of the nanoparticles. Adapted from [34] under the Creative Commons agreement from RSC, 2017.

^137^Cs^+^ adsorption in PB crystals is a very slow process. Fujita et al., have demonstrated that after two weeks of adsorption experiments, the depth of ^137^Cs^+^ adsorption was at most between 1–2 nm, irrespective of the crystal size [33]. This implies that most of the adsorption occurs on the surface of the crystallites. This low diffusion depth is mainly attributed to the blocking of the vacancies by captured ^137^Cs^+^ ions, which in turn hinders further ^137^Cs^+^ diffusion. Since the diffusion depth appears to be a constant, increasing the specific surface would therefore be a solution to increasing the ^137^Cs^+^ uptake. One way of augmenting the specific surface is by synthesizing nanoparticles of PB. The surface to volume ratio of crystallites increases as their size decreases; therefore nanoparticles have an extremely large surface to volume ratio. For example, a 3 nm nanoparticle will have 50% of its atoms on its surface. This would also imply that in the case of PB nanocrystals most of the vacancies and sites responsible for ^137^Cs^+^ adsorption would be available on the surface, thus enhancing its specific surface. To this end, different research groups have produced various PB analogs of type Metal(M)-Co, where the nature of M defines the efficiency of the uptake. Liu et al., have demonstrated that Zn assisted Fe-Co PB analogs present high ^137^Cs^+^ uptake efficiency [34]. They also observed that the size of the PB analog particle reduced with the reduction of Fe in the structure; for pure Zn-Co analogs, a crystallite size of ~73 nm was calculated, displaying the highest ^137^Cs^+^ adsorption as depicted in Figure 3. 

**Figure 4:**
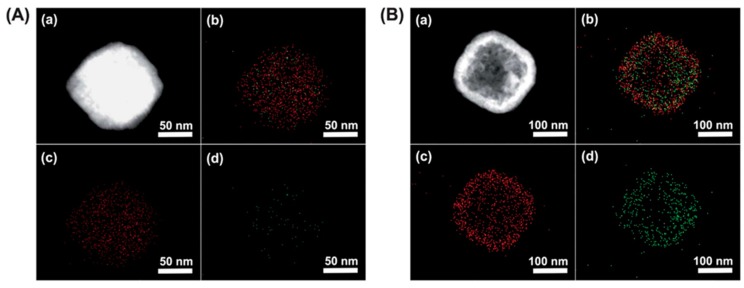
Elemental mapping images of solid (**A**) and hollow PB (**B**) nanoparticles of 190 nm in diameter. (**a**) Dark-field TEM image, (**b**) elemental mapping of both Fe and Cs (**c**) elemental mapping of Fe, and (**d**) elemental mapping of Cs. Adapted with permission from [35], Copyright RSC, 2012.

Considering that the ^137^Cs^+^ adsorption depth is only about 1–2 nm, hollow PB nanoparticles may offer many more advantages. They not only have a very active surface area due to their large surface to volume ratio but their hollow interior is also capable of capturing and storing ^137^Cs^+^ [35]. A surfactant polyvinylpyrrolidone (PVP) was used to stabilize the nanoparticle and increase their dispersion in aqueous solutions. Figure 4 compares the efficiency of solid and hollow PB cubes of ~200 nm, in the capture of ^137^Cs^+^. The elemental mapping of Figure 4B depicts a higher concentration of captured ^137^Cs^+^ ions within the hollow structures than the filled ones in Figure 4A. Nevertheless, ^137^Cs^+^ diffusion depth greater than 2 nm would require higher activation energy at room temperature. Other methods are required to determine the exact diffusion depth in such structures, as these results are mainly qualitative. Besides, it is well known that the use of a surfactant shields the active sites and prevents the capture of ^137^Cs^+^. In order to avoid such shielding effects, PB could be coated onto support materials instead through hydroxyl bonds that anchor the PB to the support material. Carboxylic groups also tend to immobilize the PB particles in a sturdier manner. Wi et al., used a polyvinyl support surface functionalized with acrylic acid. This allowed converting the OH groups to COOH and providing a better adhesion of the PB [36]. The PB nanoparticles were immobilized on the PVP sponge and an increase in ^137^Cs^+^ uptake efficiency by five times was reported, compared to the hydroxyl bond functionalization.

## 3. Magnetic Extraction Using Prussian Blue

There are reports of photo-induced magnetism where an electromagnetic radiation induces a residual magnetization even after the excitation is turned off, [37] due to low and high spin combinations of the transition metals in PB analogs. On their own PB and its analogs exhibit ferromagnetism at a Curie temperature of 11 K with a saturation magnetization of 3.4 emu/g as obtained by Tokoro et al., for Mn-Rb-Fe PB analogs [38]. The presence of Mn in the structure creates a Jahn-Teller distortion [39] by changing the M–CN–M bond angle and deviating it from 180°, whereupon inducing ferromagnetism. Among the various PB analogs, Mn based ones have shown the highest saturation magnetization [40]. One method of decreasing the Curie temperature of PB is by synthesizing nanoparticles of PB. Uemura et al., have demonstrated a decrease in Tc from 5.5 K in bulk PB to 4 K for PB nanoparticles protected by PVP [41]. Nevertheless, finding practical applications involving magnetic extraction would require having a Tc at around room temperature. Also, humidity increases the Curie temperature for Co-Cr PB analogs, [38] thus making it difficult for their direct application in aqueous media. This implies that most methods using PB for ^137^Cs^+^ extraction, do not prescribe any efficient approach to recover the exhausted adsorbent. 

Literature on nanostructured PB alone is very scarce as they are generally combined with magnetic nanomaterials like superparamagnetic iron oxide nanoparticles (SPIONs) i.e., Fe_3_O_4_ or γ-Fe_2_O_3_ nanoparticles. In the past, there have been reviews briefly describing ^137^Cs^+^ adsorption employing magnetic PB-Fe_3_O_4_ nanoparticles [16]. However, this review goes further as it not only describes more recent developments in the latter but also discusses the development of the magnetic nanocomposite in detail from a nanoscale point of view. In nanostructures, physical properties such as magnetic moment as well as adsorption vary as a function of the nanoparticle size and further depend upon the surfactants used to stabilize them during synthesis. In the paragraphs that follow, the efficiency of magnetic PB nanoparticles and their combination with carbon allotropes are assessed. To the best of our knowledge, in the literature such nanostructures have not been evaluated in detail.

**Figure 5:**
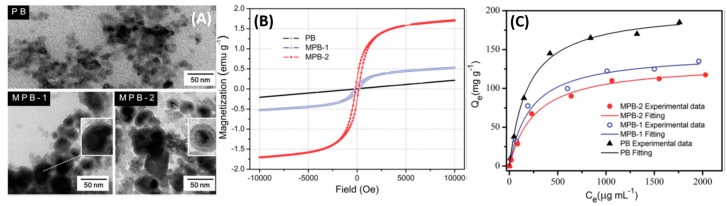
(**A**) TEM images of PB and magnetic PB (MPB) nanoparticles. (**B**) Field-dependent magnetization of PB and magnetic PB nanoparticles. (**C**) Adsorption capacity of samples under different Cs^+^ concentration. *T* = 25 °C; contact time = 6 h; [Cs^+^]_initial_ = 50–2500 μg/mL; *m*_adsorbent_/*V*_solution_ = 4 μg/mL. Adapted with permission from [44], Copyright RSC, 2016.

Core-shell structures with the magnetite constituting the core and the PB active layer constituting the shell have been employed. Jang et al., have reported that the poly(diallyldimethylammoniumchloride) (PDDA)@Iron oxide nanoparticles can act as nucleation sites for the precipitated PB, resulting in the coating of a negatively charged PB on the PDDA@Iron oxide nanoparticle surface [42]. Furthermore, they have also studied the magnetic properties of Fe_3_O_4_ and have observed a decrease in the saturation magnetization from 56 emu/g for pure Fe_3_O_4_ to 12 emu/g for the PB-Fe_3_O_4_ nanocomposite. The reduction was mostly due to the shielding of the superparamagnetism of Fe_3_O_4_ by the PB capping. Nevertheless, successful magnetic extraction was achieved with the nanocomposite [43]. PB analog compounds tend to show degradation in various applications after successive cycles of reuse. Chang et al., Figure 5A, have synthesized Fe_3_O_4_ (shell)-PB (core) nanocomposites with sizes between 20–40 nm; [44] two different concentrations of FeCl_3_ were used during the synthesis. The higher Fe concentrations produced Fe_3_O_4_ cores with a higher magnetic saturation moment (Figure 5B), which conversely had an adverse effect on the ^137^Cs^+^ adsorption due to the higher shielding of the active sites in the PB core as depicted in Figure 5C.

Some researchers have also used different ferrimagnetic nanoparticles like CoFe_2_O_4_ combined with PB for the extraction of cesium and have indicated very high efficiencies compared to pure CoFe_2_O_4_, which has some interesting ^137^Cs^+^ adsorption capacity by itself [45]. CoFe_2_O_4_ possesses the spinel structure and tends to be physically more robust but is a hard magnetic compound compared to Fe_3_O_4_. The latter is a soft magnet with a very small residual magnetization. 

## 4. Nanostructured Carbon Allotropes in ^137^Cs^+^ Capture

Carbon based materials have a large surface area with good ion-exchange capabilities. These are further enhanced by the presence of functional groups such as hydroxyl or carboxyl groups on their surfaces, which can trap heavy metals from aqueous solutions. Carbon has various allotropes such as fullerenes, graphite, and diamond. The 2D carbon allotropes include carbon nanotubes and graphene [46]. The latter is the building block for graphite, which consists of stacked graphene sheets. On the other hand, a carbon nanotube (CNT) is a rolled-up graphene sheet and belongs to the fullerene or C60 family. The number of walls on a CNT is determined by the number of times the graphene sheet is rolled-up. The defects in graphene include folds and wrinkles operating as active sites towards their decoration. In a CNT, the edges, bends and breaks in the walls promote the attachment of functional groups that are subsequently linked to other organic or inorganic nanoparticles. Therefore, CNT and graphene are attractive nanoscale structures with large surface areas displaying high ion adsorption capacities and constituting versatile catalytic supports with tailored properties. 

There are a few works based on activated carbon or carbonaceous materials in the extraction of low level ^137^Cs^+^ contamination [47]. Mesoporous carbon based magnetic nanoparticles for ^137^Cs^+^ sequestration are also being studied [48]. However, activated carbon is more efficient in extracting organic species than metal ions, [49] with an estimated adsorption efficiency between 0–10% for inorganic contaminants [50]. Activated carbon and PB analogs have shown moderate efficiency in ^137^Cs^+^ extraction with a Q_max_ = 63 mg/g [51]. PB acts as the active material while as activated carbon is the support material for the PB analog. On the other hand, nanoscale carbon-based materials are gaining importance as they can extract heavy metal ions as well as radioactive cesium ions on their own. PB and their analogs also show a higher efficiency and a greater adsorption capacity towards ^137^Cs^+^ when combined with carbon-based nanomaterials. Table 1 lists the various nanocomposites of CNT/graphene used in ^137^Cs^+^ extraction from aqueous solution. All of these nanocomposites are discussed in the paragraphs that follow.

### 4.1. Graphene Based Nanocomposites for ^137^Cs^+^ Extraction

**Figure 6:**
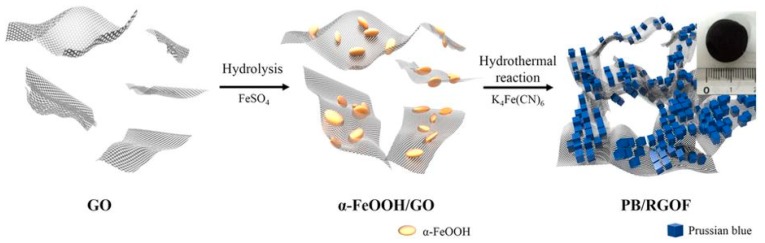
Schematics of PB and reduced graphene oxide nanocomposite synthesis in order to prepare foams. Adapted from [57] under Creative Commons agreement from Nature Research, 2015.

There is not much work on pristine graphene in the adsorption of ^137^Cs^+^ from aqueous solutions. Only one paper reporting a very high efficiency of ^137^Cs^+^ extraction using pristine graphene has been published [52]. Graphene oxide and graphene functionalized with polyaniline (PANI) have also been used in radionuclide extraction, including ^137^Cs^+^ from aqueous solutions with the former showing a lower Q_max_ than the latter [53,54]. Combining graphene with nano PB further increases the efficiency as described below. Other combinations of graphene and PB with other bioabsorbants are also available in the literature viz. chitosan and biopolymers such as pectins [55,56]. For practical application, reclaiming the exhausted nanomaterials is an important aspect to consider. In addition, once the nano PB sorbent is exhausted it takes a very long time for a small quantity of it to precipitate, as PB has a tendency to disperse like a colloid in aqueous solutions. Combining them with graphene nanosheets cannot really solve the problem of recovering the exhausted materials either, as they too tend to form a colloidal suspension in aqueous solutions depending upon the pH. One way of overcoming the problem of reclaiming these PB nanoparticles is to pack them in a graphene oxide foam (Figure 6) that could be loaded directly into the filtration column [57]. This has already been applied to organic dyes such as methylene blue where wood was impregnated with graphene, intended for a continuous flow system. The sorbent showed potential of being regenerated and was reused multiple times [58].

As mentioned earlier, PB nanoparticles have a tendency to agglomerate because of the magnetic moments generated by the various oxidation states of the transition metals [40]. In general, nanosize materials tend to agglomerate in order to reduce their surface energy [59]. The superparamagnetic properties of Fe_3_O_4_ allow easy recovery of the PB from the aqueous solution when combined together to form Fe_3_O_4_-PB nanocomposites. Nevertheless, the ^137^Cs^+^ uptake efficiency of such nanocomposites tends to decrease with a higher magnetization of Fe_3_O_4_. This is mainly because when the magnetization increases, magnetic nanoparticles tend to show higher agglomeration, which conversely reduces their active surface area. To this end, PVP surfactant was employed, which increases the mono-dispersion of the nanomaterials and provides larger surface areas for adsorption. However, surfactants tend to reduce the adsorption efficiency as seen previously. In this regard, graphene based nanocomposites are therefore an alternative to the above-mentioned shortcomings. Firstly, Fe_3_O_4_ nanoparticles are immobilized on the graphene surface in a dispersed manner, whereby reducing their agglomeration. Secondly, these nanoparticles serve as spacers between the graphene sheets discouraging the graphene sheets to stick together due to Van der Waals interactions [60]. Thirdly, PB is then directly decorated onto the graphene without being linked to the Fe_3_O_4_ nanoparticles. Here again, PB hosts the active adsorption sites whereas Fe_3_O_4_ aids in magnetically extracting the exhausted nanocomposites. Graphene mainly serves as a support even though its synergistic interaction with PB cannot be neglected. Yang et al., obtained the nanocomposite by wet impregnation of graphene with FeCl_3_ followed by K_4_[Fe(CN)_6_] with constant magnetic stirring for 1 h at room temperature. The precipitates were subsequently recovered with a magnet by them [61].

### 4.2. Carbon Nanotubes for ^137^Cs^+^ Extraction

Similarities in adsorption exist between graphene and CNT despite their opposite extreme aspect ratios. Like in the case of graphene, CNT also promises exciting ^137^Cs^+^ extraction properties. Both have the capacity to be used either as colloids that can be magnetically extracted or as membranes which allow reclaiming them easily once exhausted [62]. The mechanism of adsorption is similar to graphene and relies on electrostatic, hydrogen and π-π interactions between the carbon allotrope and the contaminant [63]. Moreover, CNT have an additional advantage compared to graphene, which is the capacity to create mesopores when bundled together. Therefore, it presents several more sites for the adsorption of ions: within the walls of the CNT, between CNT or within the mesopores, on the walls of the CNT and in peripheral grooves [64].

In addition, nitric acid treated CNT have demonstrated efficacy in the extraction of heavy metals ions such as Cu^2+^, Mn^2+^, Co^2+^, Zn^2+^, Pb^2+^ owing to amine groups on the surface. This has motivated researchers to employ CNT for the extraction of other radioactive ions from aqueous solutions viz., U^4+^, U^6+^, Th^4+^ and radioactive Co^2+^ and Cu^2+^ ions [65]. Many groups have therefore carried out treatment or functionalization of CNT in order to make them more reactive to ^137^Cs^+^ species. Several studies have shown that ^137^Cs^+^ is attracted to OH groups [66]. CNT easily harbor different groups such as amine, hydroxyl, lactone, phenol or carboxyl groups on their surfaces post-functionalization [67]. They therefore are ideal candidates for the adsorption of ^137^Cs^+^. Yang et al., have therefore prepared chitosan grafted CNT owing to the abundance of OH groups present in the former [68]. Their study demonstrated that the pH of the aqueous solution had a very important role to play on the ^137^Cs^+^ adsorption by the OH groups of chitosan. They also manifested a higher adsorption than the bare CNT. Another study determined that increasing the amount of hydroxyl groups does not necessarily bring about an increase in ^137^Cs^+^ adsorption. To this end, Yang et al., combined CNT with bentonite which are both known ^137^Cs^+^ adsorbents linked to each other via chitosan [69]. They demonstrated that cation exchange is more effective than hydroxyl capture. More importantly, they established that hydroxyl capture depends upon the host matrix and increasing the number of OH groups will not increase the ^137^Cs^+^ adsorption. This mainly suggests that the OH groups should be linked directly to the CNT matrix and are therefore limited by the number of active sites on the CNT walls. In this regard, single-walled carbon nanotube (SWCNT) could be more effective [70] than multi-walled carbon nanotube (MWCNT) [71], considering that the former possess a higher specific surface. In any case, the type of functional group also plays a role in ^137^Cs^+^ selectivity.

**Figure 7:**
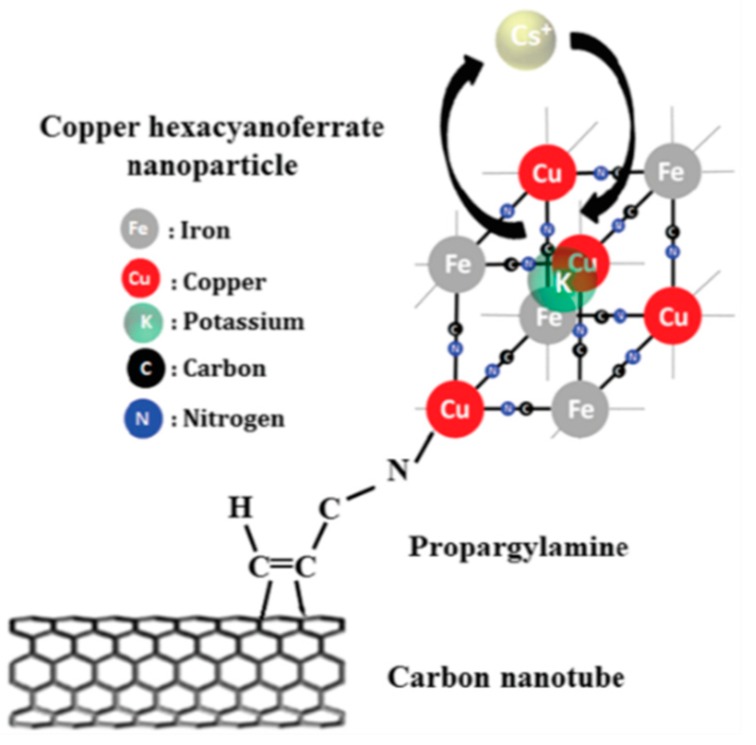
Mechanism of ^137^Cs^+^ capture in propargylmine functionalized SWCNT decorated with copper hexanoferrates. Adapted with permission from [74], Copyright RSC, 2017.

**Figure 8:**
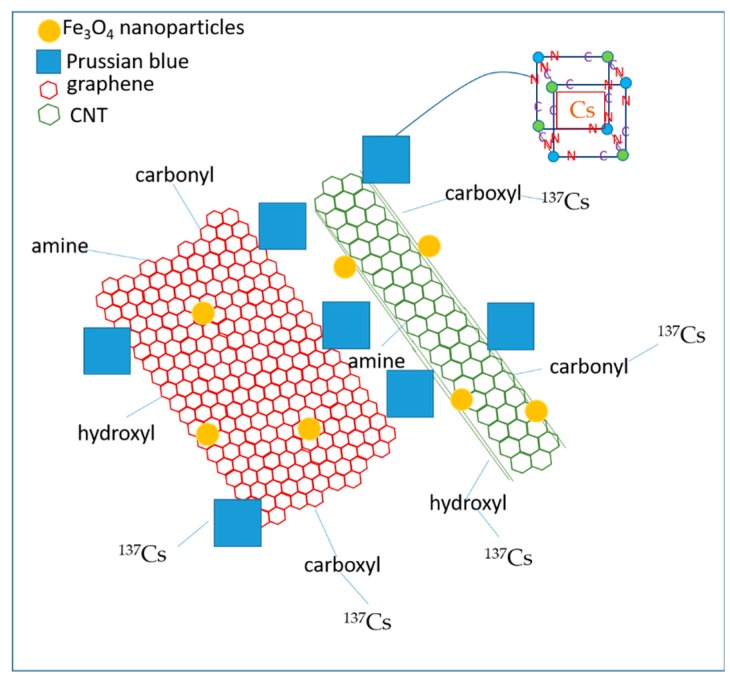
Mechanism of ^137^Cs^+^ capture in graphene/CNT/PB nanocomposite. Fe_3_O_4_ is used for magnetic extraction and does not participate in the adsorption of ^137^Cs^+^. PB is linked to CNT and graphene. Some of the possible functional groups that capture ^137^Cs^+^ are depicted on the CNT and graphene surfaces. A sub-cube or unit-cell of PB capturing and sequestering the ^137^Cs atom is also provided in the top right corner. The nanocomposite therefore harbors several active sites for the capture of ^137^Cs^+^ ions.

Other groups have studied the effects of Prussian blue and their analogs on the adsorption efficiency of ^137^Cs^+^ when combined with CNT. These tend to have a catalytic effect on the PB analogs by increasing the adsorption efficiency of the latter [72]. In one study by Li et al., Cu-Co-Ni PB analogs were combined with CNT [73]. Chitosan was wrapped around the CNT to increase their dispersion and also to link them to the PB analogs. Two studies have combined CNT with Cu cyanoferrates linked via different amine groups i.e., propargylamine (Figure 7) and dietheleneamine on SWCNT [74] and MWCNT [75], respectively. The Q_max_ increased from 150 mg/g (MWCNT) to 239 mg/g (SWCNT). These differences could be attributed to the use of different types of nanotubes and to variety of the amine ligands. In any case, the SWCNT study demonstrated that only 30% of ^137^Cs^+^ ions were adsorbed by the PB nanoparticles. The rest were adsorbed onto other active sites of the functionalized SWCNT. Tsuruoka et al., have studied ferrocyanides embedded into diatomite nanoparticles encaged in both MWCNT and SWCNT which were impregnated in a polyurethane spongiform [76]. They have observed a synergistic effect owing to the porous structures created by both types of CNT. Zheng et al., have also demonstrated that it was possible to electrochemically clean the nanocomposite of ^137^Cs^+^ ions and reuse them [77]. Water-soluble sodium CoHCF encapsulated in alginate beads reinforced with highly dispersed CNT was employed in the extraction of ^137^Cs^+^. The beads were stable in a broad pH range of 4–10 and revealed potential for large scale applications [72]. A combination of graphene, carbon fiber and PB has also been used to extract ^137^Cs^+^ directly from a lake in China [78]. Such a combination therefore allows an increased specific surface area with prospective scalable applications. A high efficacy of the graphene-CNT nanocomposites has been demonstrated for the extraction of aromatic compounds and Cu^+2^ heavy metal ions [79]. These hybrid carbon allotropes exhibit 25% higher adsorption capacities in both cases than their individual counterparts. They have also shown high desalination capacities along with adsorption of heavy metal ions [80]. This suggests that alkali metal ions would tend to have a higher affinity towards the hybrid carbon allotrope compared to PB, making ^137^Cs^+^ extraction by PB more effective in a high salinity aqueous medium such as seawater. Combining them with Fe_3_O_4_ would then be a suitable method of reclaiming the exhausted sorbents. Figure 8 provides the schematic of the ^137^Cs^+^ extraction mechanism of graphene/CNT/magnetic PB nanocomposites.

## 5. Efficiency of Various Carbon Based Materials in ^137^Cs^+^ Capture

**Figure 9:**
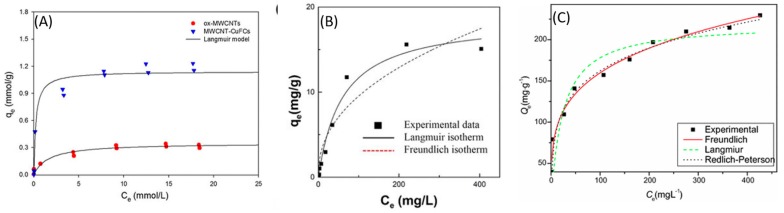
Examples of Langmuir, Freundlich and Redlich-Peterson adsorption isotherms models applied to (**A**) oxidized MWCNT and CuFC-MWCNT, adapted with permission from [75], Copyright Springer Nature, 2015, (**B**) Graphene foam-PB composite, adapted from [57] with permission under Creative Commons agreement from Nature Research, 2015 and (**C**) Chitosan-PB analog-CNT, adapted with permission from [73], Copyright Springer Nature, 2016.

Various batch tests performed on the different sorbents have shown increased efficiency of the graphene or CNT based sorbents (Table 1). Adsorption kinetics and equilibrium studies provide the efficacy of an adsorbent in a given adsorption system and are necessary to understand the underlying adsorption mechanisms. Most of the equilibrium adsorption studies that are provided in Table 1, employ the Langmuir and Freundlich adsorption models [67,77]. In the Langmuir model, the adsorption of ^137^Cs^+^ from an aqueous solution onto a graphene-based nanocomposite is mainly used to obtain Q_max_ values. The model would hold for graphene-based nanocomposites as only a single monolayer of adsorption is considered, as defined by the active sites on the graphene/CNT surfaces. In the Langmuir model, all sites are considered equally favorable. This is contrary to the Freundlich model, which considers that the active sites possess varying adsorption energies and such is the case of graphene-PB based nanocomposites. In the Tempkin model it is assumed that the adsorption energy reduces as and when the sites become saturated [71,77]. The Brunauer-Emmet-Teller model assumes multilayer adsorption by considering the Langmuir model for each adsorbed layer [75]. Figure 9 provides examples of some of the isotherm models used in CNT/graphene-PB nanocomposites surveyed in this work. However, graphene and CNT based PB nanocomposites can be regarded as monolayer adsorption considering that the active sites lie on the surface of CNT and graphene, implying that all three models, Langmuir, Freundlich and Tempkin are appropriate. These equilibrium models are seconded by kinetic models such as, the pseudo first order (PFO) and second order kinetic models (PSO), which define the adsorption rate limiting factors [70,78,81,82]. The Elovich kinetic model has also been applied to chitosan-CNT-PB composites in consideration of their elemental heterogeneity, as the solid surfaces under study are energetically heterogeneous also [74].

Table 1 below therefore provides Q_max_ values obtained from batch tests of several different CNT, graphene, Fe_3_O_4_ and PB combinations. Q_max_ gives a direct indication of the available active sites or the maximum monolayer capacity for ^137^Cs^+^ adsorption. Moreover, the Langmuir equation is valid over a wide concentration range. The table indicates that PB analogs along with CNT statistically provide the highest Q_max_ values reaching 310 mg/g. Pristine CNT or graphene in general do not show much efficiency compared to their hybrid counterparts. However, when they are decorated with amine groups their Q_max_ reaches 240 mg/g. This value is higher than CNT attached with hydroxyl groups of chitosan. However, chitosan when used as a linker between CNT and PB has a Q_max_ of 219 mg/g. Nevertheless, the value of the Q_max_ depends on various factors such as pH, temperature of the solution, presence of competing ions and the initial concentration of ^137^Cs^+^. The particle size of the sorbent is also an important consideration but since we are only considering nanosize sorbents in this review, we can neglect this parameter in our discussions. Various studies on graphene or CNT based materials in this study show that the temperature of the solvent is instrumental in increasing the Q_max_ of the sorbent [54,71,78]. This is mainly because the process becomes endothermic with temperature increase and ^137^Cs^+^ in the solution is therefore more mobile due to which they attain active sites more efficiently on the sorbent. This further implies that ^137^Cs^+^ capture efficiency and can increase if the number of active sites obtained by various treatment or functionalization on the carbon-based materials are increased to their maximum capacity. The adsorption of ^137^Cs^+^ is also highly dependent on the initial pH of the solution, which has mostly to do with the functional groups of the carbon-based materials becoming protonated at low pH [67,72]. Since an OH group is necessary for the capture of ^137^Cs^+^ as discussed above a reduction in the former due to protonation would decrease the Q_max_ of the sorbent. At higher pH, a deprotonation of the functional group and an increase in OH^-^ ions in the solution makes the ^137^Cs^+^ capture process more efficient.

The presence of other ions or salts such as Mg, Na, Li and K also affects the ^137^Cs^+^ adsorption due to the inherent competition between them. They all manifest similar affinities to the active sites on the carbon-PB based nanocomposites. In the case of PB or its analog, a higher affinity to K^+^ and Na^+^ has been observed with a given order of competition: K^+^ > Ca^2+^ > Mg^2+^ > Na^+^. When combined with MWCNT, the Q_max_ value decreased only slightly but showed a higher selectivity towards ^137^Cs^+^ [75]. Lin et al., have used CNT-PANI-NiHCF composites for electrically switching ion exchange studies and have demonstrated a higher affinity towards ^137^Cs^+^ than Na^+^ ions [83]. Such nanocomposites are also employed for the extraction of Sr^2+^ at the same time. However, they appear to have a greater affinity towards ^137^Cs^+^ than ^81^Sr^2+^, which may have to do with the difference of oxidation states of the two cations [73]. The contact time between the sorbent and the sorbate are also necessary parameters towards understanding the adsorption of ^137^Cs^+^. Optimal adsorption times are required in order to evaluate the feasible scalability of the developed methods. This means that the contact time should be calculated in such a way that optimum adsorption should be obtained with minimum leaching of the adsorbed ^137^Cs^+^ as well as the transition metals and alkali metals constituting the PB framework. Both adsorption and equilibrium times should be short for large-scale applications. The rate of adsorption depends on the temperature, number of adsorption sites, and diffusion of the ^137^Cs^+^ within the porous matrix, which in turn depends on the density of the packed material. In the case of MWCNT, a contact time of 80 min was required to attain equilibrium [67]. A study with graphene oxide required a long contact time of 24 h [54] similarly to stirred pectin stabilized magnetic PB-GO nanocomposites [56]. For PB-graphene-carbon fiber composite the equilibrium was reached after 8 h [78].

## 6. Toxicity of Nanomaterials

Toxicity of nanomaterials is a key issue that is under debate [84]. If used in water remediation systems, their release and disposal need to be properly planned and evaluated. Nanomaterials are small enough to pass the skin barrier and enter the blood stream. In addition, they can be inhaled causing lung damage or ingested causing kidney damage. In the case of heavy metals, their ions can be fatal and can engender life-threatening maladies. Both CNT and graphene have been studied to evaluate their toxicity and environmental impact. Studies on zebra fish have shown that both their growth and cardiac rates are affected by these 2D nanocarbon allotropes [85]. The toxicity of CNT is an important enough issue to incite the development of related counter small-molecule-drugs [86]. CNT present risks during their entire lifecycle and mainly during occupational exposure. This implies that their manipulation needs to be regulated. CNT could also interact with biomolecules in the water and produce toxic effects [87]. The stability of PB needs to be thoroughly evaluated in various aqueous conditions. Studies have demonstrated the possibility of leaching of cyanide into ground water on decomposition of the ferrocyanides [88]. This also implies that ^137^Cs^+^ could leach out and contaminate ground water. One method to stabilize PB analogs viz., titanium ferrocyanide and curb leaching of cyanide and ^137^Cs^+^ includes transforming the spent material into lithium titanate, which consequently immobilizes the ^137^Cs [89]. In some studies, the release of iron from PB in Fenton type reactions were studied by Doumic et al. [90]. They studied the catalytic effect in a pH = 3 environment and have observed that insoluble PB nanocomposites were more stable and showed negligible Fe release (10% after 13 cycles). Kim et al., also have observed negligible leaching of Fe during the ^137^Cs^+^ adsorption process in PB-cellulose hydrogel composites [91]. Yang et al., have also carried out a systematic study of the possible leaching of Fe from PB-Fe_3_O_4_-graphene nanocomposites in a wide pH range from 4 to 10 [61]. Even in the highest ionic strength seawater, the amount of Fe leaching ranged from 0.95% to 0.61% while in natural water, Fe leaching as low as 0.0026% was observed. In another study with PB embedded magnetic hydrogels, leaching of Fe was studied over a period of two weeks and only a 0.3% increase in Fe was observed in the solution [81]. Graphene is also considered a chemically stable material. Nevertheless, changes in pH affect the protonation of the carbonyl or hydroxyl groups. At high pH graphene dissolves like a salt while at low pH, it tends to form aggregates and at neutral pH it stays suspended in the solution [92]. This also implies that the stability of graphene oxide depends upon the functional groups on its surface and their particle sizes [93]. In such a scenario, the dose related toxicity could vary. Overall, the formation of colloids should be absolutely avoided. However, the effect of graphene on the human body is still unknown; on the other hand, graphene oxide has been known to accumulate in the lungs of mice when inhaled, but no pathological outcome was further reported. Nano-graphite and Fe_3_O_4_ were also tested for Fe leaching. A very low pH < 3 showed an exponential increase of Fe release. Fe_3_O_4_ are linked to graphene via electrostatic forces or via functional groups on their surfaces similar to CNT therefore, the risk of nanoparticles being loosely bonded does exist. Nevertheless, in general, many studies have shown that linking Fe_3_O_4_ to graphene and CNT exhibit exceptionally high stability with very little leaching of Fe [94]. In any case, the exhausted nanoadsorbent has to be removed from the water and presently; magnetic extraction appears to be the most adapted solution.

## 7. Conclusions and Perspectives

### 7.1. The Road so Far for ^137^Cs^+^ and the Prospects of Graphene/CNT-PB Based Nanocomposites Towards Its Extraction

Research on ^137^Cs^+^ contamination has taken giant leaps since the Chernobyl catastrophe. Cesium is known to damage soft tissue, bones and provoke bone cancer. Today, eight years after the Fukushima Daiichi disaster, cesium still seems to be prevalent in soil and water; a large-scale cleanup at both fronts is still being pursued. Radioactive ^137^Cs^+^ has also been detected in tap waters of Tokyo. Salty ground water containing ^137^Cs^+^ is one artery for land contamination to reach the sea. Even though there is some on-site storage capacity at Fukushima Daiichi of the contaminated water, a large amount of the least contaminated water has already been rejected into the sea. There has been some progress in the cleansing of waters, however the problem is still at large. Tokyo Electric Power Company (TEPCO) forecasts the decontamination procedure to continue for the next 30–40 years. Therefore, new materials and methods towards the capture and sequestration of ^137^Cs^+^ are being developed at an accelerated pace in order to quickly reply to the concern. 

Adsorption and ion exchange have been found to be efficient methods in the capture of ^137^Cs^+^. The contamination in Fukushima has been cleaned by the Kurion process, which consisted of effective capture of the ions (^81^Sr^2+^ and ^134,137^Cs^+^) via an extremely porous aluminosilicate based zeolite [95]. Areva, the French nuclear company then further treated the water with a nickel ferrocyanide (NiHFC) and sand-polymer mixtures [96], which reduced the contamination by at least more than 1000 times. Advanced liquid processing system (ALPS) technology uses different combinations of resins, zeolites, titanates, activated carbons and hexacyanoferrates with a selectivity towards 62 radionuclides but is only applicable after ^137^Cs, ^134^Cs and ^90^Sr quantities are reduced by the Kurion and Areva methods [97]. Therefore, the Prussian blue analog or hexacyanoferrates have already shown efficiency at a large scale and a total of 482,000 m^3^ of water has been decontaminated so far. The major drawback of the methods was the similarity between Na^+^ and ^134,137^Cs^+^ affinities thus, making the whole process less efficient. The storage of sludge obtained via such processes was initially planned in large underground tanks on the Fukushima-Daiichi premises with no radioactive leaching detected. The waste was then immobilized by cementation treatment [98]. The major disadvantage of cementation was the increase in volume of the waste to be stored in a nuclear repository along with the risk of the cement cracking. To this end, vitrification has been proposed as a method that not only immobilizes ^137^Cs more securely without the risk of breaking or cracking but also compresses the quantity of stored radioactive waste.

Graphene based nanomaterials seem to have a catalytic effect on PB as the Na^+^ and K^+^ selectivity decreases compared to that of ^137^Cs^+^. This selectivity towards ^137^Cs^+^ can be further enhanced on electrical switching as in the case of PB with CNT. In addition, ^81^Sr^2+^ and ^134^Cs^+^ ions are also extracted simultaneously with ^137^Cs^+^. Nevertheless, PB does show a higher affinity to ^134,137^Cs^+^ than ^81^Sr^2+^, which can be further enhanced by using high specific surface materials such as graphene, CNT or hollow nano PB and their analogs, which exhibit higher Q_max_ values than their bulk counterparts. These nanopowders can be dispersed into a water body to be decontaminated and recovered via magnetic extraction on attaching Fe_3_O_4_ nanoparticles to the graphene surfaces. The drawback of Fe_3_O_4_ is that the saturation magnetization is rather low for large-scale applications as they usually contain surfactants. This would imply a risk of leaving behind some nanomaterial uncaught by the applied magnetic field. Another scenario could consist of making membranes of functionalized graphene and CNT with PB, which could then be easily recovered once exhausted. The main drawback of using PB is an increase of alkali metal content in the water due to ion exchange. This would not have detrimental effects on seawater; however, increased salinity in ground water could render it unsafe to drink.

Graphene and CNT are resistant in harsh environments and further offer recycling capabilities. Moreover, carbon is the fourth most abundant element in the Earth’s crust implying continuous availability for large-scale applications. However, no publications reporting large-scale use of graphene or CNT in water filtration are available. This suggests that new technologies and methods employing them are potential candidates. Nevertheless, large-scale applications require large cost-effective production and functionalization capacities. Moreover, a choice between fixed bed and batch adsorption has to be made when employing graphene. In certain cases, batch methods appear to be more efficient for contaminant adsorption than fixed bed methods. Such a choice would allow obtaining maximal adsorption with minimal amounts of absorbent, thereby reducing the quantities of radioactive wastes to be treated [99]. Further studies on the toxicity of these carbon-based materials are also required. The most important parameter is the contact time of the adsorbent with the adsorbate, which should be as low as possible for large-scale applications especially considering the thousands of cubic meter waiting to be remediated. Regeneration of graphene and CNT has been studied using acids and bases, making the recovery of the metallic contaminants feasible. However, from an industrial point of view, there is much work that needs to be conducted in this very promising field.

### 7.2. Development of Other Nanocomposites

Different materials need to be combined in order to increase the ^137^Cs^+^ selectivity and hence its extraction efficiency, as already proven by the Kurion-Areva-ALPS combinations. With regards to other potential materials, PB has also been combined with zeolites for the adsorption of ^137^Cs^+^ ions [100,101]. Zeolites were seen as promising materials [101] for ^137^Cs^+^ adsorption [102,103] and were subsequently combined with fibrous polymer adsorbents to enhance their ^137^Cs^+^ extraction capacity. These zeolite-polymer composite fibers were initially studied for heavy metal ion removal such as Pb^2+^, Cu^2+^, Ni^2+^, and Cd^2+^ [104,105]. One advantage is that the surface to volume ratio could be tuned through the zeolite fibrous polymer ratio with a specific surface value of 145 m^2^/g. The study suggested that with such zeolite polymer composite fibers, the Pb^2+^ extraction takes place by both ion exchange and inclusion mechanisms. Zeolite and poly(ethersulfone) were then combined for ^137^Cs^+^ extraction with 30 wt% of zeolite in porous fibrous polymer [15]. These composites were applied to the decontamination of radioactive ^137^Cs^+^ in the city of Fukushima for a period of 28 days. A total of 7700 Bq/kg of radioactive Cs was extracted during this period, while with the zeolite alone only 33 Bq/kg was extracted [7]. The main advantage of this composite is that water can flow unobstructed through the composite compared to the zeolite powders. Fiber based porous structures seem to be more adapted for large flow extraction than magnetic extraction that needs a specific set-up. The latter nevertheless has the advantage of limiting the quantity of nuclear waste produced during the radioactive ^137^Cs^+^ extraction process. Functionalized graphene and CNT could very well be included in such porous structures without clogging the pores and increasing the ^137^Cs^+^ selectivity during extraction.

## Figures and Tables

**Table 1:** Various Graphene/carbon nanotubes (CNT)/PB/Fe_3_O_4_ based nanocomposites arranged in decreasing order of their Q_max_. The table provides the ^137^Cs^+^removal efficiency, *K*_d_, intitial concentration, temperature of extraction, isotherm and kinetic models used.

Nanocomposite	Removal Efficiency %	*K*_d_ L/g	pH	Initial Concentration mg/L	Temperature °C	Isotherm	Kinetic Model	Q_max_ mg/g
Pristine graphene [52]	41	0.115	1–12	10–10000	27	Langmuir	NA	465
Fe_3_O_4_-GO-pectin [56]	NA	50	1–11	9–822	30	Langmuir Freundlich Tempkin	PFO PSO	432
MWCNT-CuHCF [77]	95	568	7	1	28	Langmuir Fruendlich	NA	310
SWCNT-DMAD [70]	NA	NA	NA	NA	25	NA	NA	250
SWCNT-CuHCF [74]	NA	NA	NA	NA	25	NA	NA	230
Chitosan-PB-CNT [73]	NA	42.5	1–9	200	20	Langmuir Freundlich Redlich Peterson	PFO PSO Boyd Webbe Morris Elovich	219
GO-PANI [53]	100	94.5	2–10	1–100	25	Langmuir Freundlich	NA	185
Bentonite [69]	89	34.2	3–10	10	25	Langmuir Freundlich	NA	182.7
MWCNT-Cu ferrocyanide [75]	NA	NA	1–12	0.027–55	25	Langmuir	NA	151
MWCNT-Na-CoHCF-alginate [72]	53	23	2–10	200	25	Langmuir Freundlich	PFO PSO	133.33
MWCNT-Amino functionalized [71]	95	7–13	1–9	4.5 × 10^−6^–2.28 × 10^−4^	15–35	Langmuir Freundlich Tempkin	PFO PSO	117–136
PB-GO-Carbon fiber [78]	85.48	159.8	2–8	5–80	25	Langmuir Freundlich	PFO PSO	81.25
MWCNT [75]	NA	NA	1–12	0.027–55	25	Langmuir	NA	46
Chitosan-MWCNT [68]	25–70	26.7	3–10	1–42	25	Langmuir Freundlich	NA	45
Fe3O4-PB-GO- alginate beads [82]	80	48.7	7	25–150	0–30	Langmuir Freundlich	PSO	43.5
MWCNT-betonite [69]	80	23	3–10	10	25	Langmuir Freundlich	NA	159.4
Fe_3_O_4_-PB-hydrogels [81]	99.5	0.4	4–10	100–500	25	Langmuir	PSO	41.5
GO [54]	55	481	1–13	10	23	Langmuir Freundlich	PFO PSO	40
MWCNT [69]	35	27.8	3–10	10	25	Langmuir Freundlich	NA	45.6
Raw MWCNT [68]	12–55	55.6	3–10	1–42	25	Langmuir Freundlich	NA	29
RGO-PB [57]	99.5	6.455	NA	0.2	NA	Langmuir Freundlich	NA	18.67
MWCNT [67]	45	35	1–10	5–20	25	Langmuir Freundlich	NA	12.75
Pristine MWCNT [67]	NA	14	1–10	5–20	25	Langmuir Freundlich	NA	1.63

NA-not available.

## References

[1] Delmore J.E., Snyder D.C., Tranter T., Mann N.R. (2011). Cesium isotope ratios as indicators of nuclear power plant operations. J. Environ. Radioact..

[2] Vakulovsky S.M., Nikitin A.I., Chumichev V.B., Katrich I.Y., Voitsekhovich O.A., Medinets V.I., Pisarev V.V., Bovkum L.A., Khersonsky E.S. (1994). Cesium-137 and strontium-90 contamination of water bodies in the areas affected by releases from the chernobyl nuclear power plant accident: An overview. J. Environ. Radioact..

[3] Nesterenko V.B., Yablokov A.V. (2009). Chapter I. Chernobyl Contamination: An Overview. Ann. N. Y. Acad. Sci..

[4] Varskog P., Næumann R., Steinnes E. (1994). Mobility and plant availability of radioactive Cs in natural soil in relation to stable Cs, other alkali elements and soil fertility. J. Environ. Radioact..

[5] Morino Y., Ohara T., Watanabe M., Hayashi S., Nishizawa M. (2013). Episode Analysis of Deposition of Radiocesium from the Fukushima Daiichi Nuclear Power Plant Accident. Environ. Sci. Technol..

[6] Parajuli D., Tanaka H., Hakuta Y., Minami K., Fukuda S., Umeoka K., Kamimura R., Hayashi Y., Ouchi M., Kawamoto T. (2013). Dealing with the Aftermath of Fukushima Daiichi Nuclear Accident: Decontamination of Radioactive Cesium Enriched Ash. Environ. Sci. Technol..

[7] Kobayashi T., Ohshiro M., Nakamoto K., Uchida S. (2016). Decontamination of Extra-Diluted Radioactive Cesium in Fukushima Water Using Zeolite–Polymer Composite Fibers. Ind. Eng. Chem. Res..

[8] Staunton S., Dumat C., Zsolnay A. (2002). Possible role of organic matter in radiocaesium adsorption in soils. J. Environ. Radioact..

[9] Lieser K.H., Steinkopff T. (1989). Chemistry of radioactive cesium in the hydrosphere and in the geosphere. Radiochim. Acta.

[10] Gibert O., Valderrama C., Peterkóva M., Cortina J.L. (2010). Evaluation of Selective Sorbents for the Extraction of Valuable Metal Ions (Cs, Rb, Li, U) from Reverse Osmosis Rejected Brine. Solvent Extr. Ion Exch..

[11] Kosaka K., Asami M., Kobashigawa N., Ohkubo K., Terada H., Kishida N., Akiba M. (2012). Removal of radioactive iodine and cesium in water purification processes after an explosion at a nuclear power plant due to the Great East Japan Earthquake. Water Res..

[12] Adabbo M., Caputo D., de Gennaro B., Pansini M., Colella C. (1999). Ion exchange selectivity of phillipsite for Cs and Sr as a function of framework composition. Microporous Mesoporous Mater..

[13] Chitry F., Pellet-Rostaing S., Nicod L., Gass J.-L., Foos J., Guy A., Lemaire M. (2001). Cesium/sodium separation by nanofiltration-complexation in aqueous medium. Sep. Sci. Technol..

[14] Mahendra C., Bera S., Babu C.A., Rajan K.K. (2013). Separation of Cesium by Electro Dialysis Ion Exchange using AMP-PAN. Sep. Sci. Technol..

[15] Masaru Ooshiro T.K. (2017). Shuji Uchida Fibrous zeolite-polymer composites for decontamination of radioactive waste water extracted from radio-Cs fly ash. Int. J. Eng. Tech. Res..

[16] Liu X., Chen G.-R., Lee D.-J., Kawamoto T., Tanaka H., Chen M.-L., Luo Y.-K. (2014). Adsorption removal of cesium from drinking waters: A mini review on use of biosorbents and other adsorbents. Bioresour. Technol..

[17] Olatunji M.A., Khandaker M.U., Mahmud H.N.M.E., Amin Y.M. (2015). Influence of adsorption parameters on cesium uptake from aqueous solutions—A brief review. RSC Adv..

[18] El-Rahman K.M.A., El-Sourougy M.R., Abdel-Monem N.M., Ismail I.M. (2006). Modeling the Sorption Kinetics of Cesium and Strontium Ions on Zeolite A. J. Nucl. Radiochem. Sci..

[19] Awual M.R., Yaita T., Miyazaki Y., Matsumura D., Shiwaku H., Taguchi T. (2016). A Reliable Hybrid Adsorbent for Efficient Radioactive Cesium Accumulation from Contaminated Wastewater. Sci. Rep..

[20] Solbrå S., Allison N., Waite S., Mikhalovsky S.V., Bortun A.I., Bortun L.N., Clearfield A. (2001). Cesium and Strontium Ion Exchange on the Framework Titanium Silicate M_2_Ti_2_O_3_SiO_4_·nH_2_O (M = H, Na). Environ. Sci. Technol..

[21] Behrens E.A., Clearfield A. (1997). Titanium silicates, M_3_HTi_4_O_4_(SiO_4_)_3_·4H_2_O (M=Na^+^, K^+^),with three-dimensional tunnel structures for the selective removal of strontium and cesium from wastewater solutions. Microporous Mater..

[22] Cho Y., Seol B.N. (2013). A Study on Removal of Cesium and Strontium from Aqueous Solution Using Synthetic Na-Birnessite. J. Korean Soc. Water Wastewater.

[23] El-Naggar I.M., Mowafy E.A., El-Aryan Y.F., Abd El-Wahed M.G. (2007). Sorption mechanism for Cs^+^, Co^2+^ and Eu^3+^ on amorphous zirconium silicate as cation exchanger. Solid State Ion..

[24] Kumar A., Yusuf S.M., Keller L. (2005). Structural and magnetic properties of Fe[Fe(CN)_6_]∙4H_2_O. Phys. Rev. B.

[25] Nie P., Shen L., Luo H., Ding B., Xu G., Wang J., Zhang X. (2014). Prussian blue analogues: A new class of anode materials for lithium ion batteries. J. Mater. Chem. A.

[26] Matsuda T., Kim J., Moritomo Y. (2012). Control of the alkali cation alignment in Prussian blue framework. Dalton Trans..

[27] Lu Y., Wang L., Cheng J., Goodenough J.B. (2012). Prussian blue: A new framework of electrode materials for sodium batteries. Chem. Commun..

[28] Ishizaki M., Akiba S., Ohtani A., Hoshi Y., Ono K., Matsuba M., Togashi T., Kananizuka K., Sakamoto M., Takahashi A. (2013). Proton-exchange mechanism of specific Cs^+^ adsorption via lattice defect sites of Prussian blue filled with coordination and crystallization water molecules. Dalton Trans..

[29] Ruankaew N., Yoshida N., Watanabe Y., Nakano H., Phongphanphanee S. (2017). Size-dependent adsorption sites in a Prussian blue nanoparticle: A 3D-RISM study. Chem. Phys. Lett..

[30] Eberl D.D. (1980). Alkali Cation Selectivity and Fixation by Clay Minerals. Clays Clay Miner..

[31] Takahashi A., Tanaka H., Minami K., Noda K., Ishizaki M., Kurihara M., Ogawa H., Kawamoto T. (2018). Unveiling Cs-adsorption mechanism of Prussian blue analogs: Cs^+^-percolation via vacancies to complete dehydrated state. RSC Adv..

[32] Ayrault S., Jimenez B., Garnier E., Fedoroff M., Jones D.J., Loos-Neskovic C. (1998). Sorption Mechanisms of Cesium on Cu^II^_2_Fe_II_(CN)_6_ and Cu^II^_3_[Fe^III^(CN)_6_]_2_ Hexacyanoferrates and Their Relation to the Crystalline Structure. J. Solid State Chem..

[33] Fujita H., Miyajima R., Sakoda A.J.A. (2015). Limitation of adsorptive penetration of cesium into Prussian blue crystallite. Adsorption.

[34] Liu J., Li X., Rykov A.I., Fan Q., Xu W., Cong W., Jin C., Tang H., Zhu K., Ganeshraja A.S. (2017). Zinc-modulated Fe–Co Prussian blue analogues with well-controlled morphologies for the efficient sorption of cesium. J. Mater. Chem. A.

[35] Torad N.L., Hu M., Imura M., Naito M., Yamauchi Y. (2012). Large Cs adsorption capability of nanostructured Prussian Blue particles with high accessible surface areas. J. Mater. Chem..

[36] Wi H., Kang S.-W., Hwang Y. (2019). Immobilization of Prussian blue nanoparticles in acrylic acid-surface functionalized poly(vinyl alcohol) sponges for cesium adsorption. Environ. Eng. Res..

[37] Pajerowski D.M., Gardner J.E., Frye F.A., Andrus M.J., Dumont M.F., Knowles E.S., Meisel M.W., Talham D.R. (2011). Photoinduced Magnetism in a Series of Prussian Blue Analogue Heterostructures. Chem. Mater..

[38] Tokoro H., Ohkoshi S.-I. (2011). Novel magnetic functionalities of Prussian blue analogs. Dalton Trans..

[39] Buzin E.R., Prellier W., Mercey B., Simon C., Raveau B. (2002). Relations between structural distortions and transport properties in Nd_0.5_Ca_0.5_MnO_3_ strained thin films. J. Phys. Condens. Matter.

[40] Nakotte H., Shrestha M., Adak S., Boergert M., Zapf V.S., Harrison N., King G., Daemen L.L. (2016). Magnetic properties of some transition-metal Prussian Blue Analogs with composition M_3_[M′(C,N)_6_]_2_·xH_2_O. J. Sci. Adv. Mater. Devices.

[41] Uemura T., Kitagawa S. (2003). Prussian Blue Nanoparticles Protected by Poly(vinylpyrrolidone). J. Am. Chem. Soc..

[42] Jang S.-C., Kang S.-M., Kim G.Y., Rethinasabapathy M., Haldorai Y., Lee I., Han Y.-K., Renshaw J.C., Roh C., Huh Y.S. (2018). Versatile Poly(Diallyl Dimethyl Ammonium Chloride)-Layered Nanocomposites for Removal of Cesium in Water Purification. Materials.

[43] Jang J., Lee D.S. (2016). Magnetic Prussian Blue Nanocomposites for Effective Cesium Removal from Aqueous Solution. Ind. Eng. Chem. Res..

[44] Chang L., Chang S., Chen W., Han W., Li Z., Zhang Z., Dai Y., Chen D. (2016). Facile one-pot synthesis of magnetic Prussian blue core/shell nanoparticles for radioactive cesium removal. RSC Adv..

[45] Hassan M.R., Aly M.I. (2019). Adsorptive removal of cesium ions from aqueous solutions using synthesized Prussian blue/magnetic cobalt ferrite nanoparticles. Part. Sci. Technol..

[46] Nasir S., Hussein M.Z., Zainal Z., Yusof N.A. (2018). Carbon-Based Nanomaterials/Allotropes: A Glimpse of Their Synthesis, Properties and Some Applications. Materials.

[47] Kimura K., Hachinohe M., Klasson K.T., Hamamatsu S., Hagiwara S., Todoriki S., Kawamoto S. (2014). Removal of Radioactive Cesium (^134^Cs plus ^137^Cs) from Low-Level Contaminated Water by Charcoal and Broiler Litter Biochar. Food Sci. Technol. Res..

[48] Husnain S.M., Um W., Chang Y.-Y., Chang Y.-S. (2017). Recyclable superparamagnetic adsorbent based on mesoporous carbon for sequestration of radioactive Cesium. Chem. Eng. J..

[49] Sweetman M.J., May S., Mebberson N., Pendleton P., Vasilev K., Plush S.E., Hayball J.D. (2017). Activated Carbon, Carbon Nanotubes and Graphene: Materials and Composites for Advanced Water Purification. C.

[50] Brown J., Hammond D., Wilkins T. (2008). Handbook for Assessing the Impact of a Radiological Incident on Levels of Radioactivity in Drinking Water and Risks to Operatives at Water Treatment Works: Supporting Scientific Report.

[51] Wang L., Feng M., Liu C., Zhao Y., Li S., Wang H., Yan L., Tian G., Li S. (2009). Supporting of Potassium Copper Hexacyanoferrate on Porous Activated Carbon Substrate for Cesium Separation. Sep. Sci. Technol..

[52] Kaewmee P., Manyam J., Opaprakasit P., Truc Le G.T., Chanlek N., Sreearunothai P. (2017). Effective removal of cesium by pristine graphene oxide: Performance, characterizations and mechanisms. RSC Adv..

[53] Sun Y., Shao D., Chen C., Yang S., Wang X. (2013). Highly Efficient Enrichment of Radionuclides on Graphene Oxide-Supported Polyaniline. Environ. Sci. Technol..

[54] Tan L., Wang S., Du W., Hu T. (2016). Effect of water chemistries on adsorption of Cs(I) onto graphene oxide investigated by batch and modeling techniques. Chem. Eng. J..

[55] Rethinasabapathy M., Kang S.-M., Lee I., Lee G.-W., Lee S., Roh C., Huh Y.S. (2019). Highly stable Prussian blue nanoparticles containing graphene oxide–chitosan matrix for selective radioactive cesium removal. Mater. Lett..

[56] Kadam A.A., Jang J., Lee D.S. (2016). Facile synthesis of pectin-stabilized magnetic graphene oxide Prussian blue nanocomposites for selective cesium removal from aqueous solution. Bioresour. Technol..

[57] Jang S.-C., Haldorai Y., Lee G.-W., Hwang S.-K., Han Y.-K., Roh C., Huh Y.S. (2015). Porous three-dimensional graphene foam/Prussian blue composite for efficient removal of radioactive ^137^Cs. Sci. Rep..

[58] Goodman S.M., Bura R., Dichiara A.B. (2018). Facile Impregnation of Graphene into Porous Wood Filters for the Dynamic Removal and Recovery of Dyes from Aqueous Solutions. ACS Appl. Nano Mater..

[59] Laurent S., Forge D., Port M., Roch A., Robic C., Vander Elst L., Muller R.N. (2008). Magnetic Iron Oxide Nanoparticles: Synthesis, Stabilization, Vectorization, Physicochemical Characterizations, and Biological Applications. Chem. Rev..

[60] Devasenathipathy R., Mani V., Chen S.-M., Arulraj D., Vasantha V.S. (2014). Highly stable and sensitive amperometric sensor for the determination of trace level hydrazine at cross linked pectin stabilized gold nanoparticles decorated graphene nanosheets. Electrochim. Acta.

[61] Yang H., Sun L., Zhai J., Li H., Zhao Y., Yu H. (2014). In situ controllable synthesis of magnetic Prussian blue/graphene oxide nanocomposites for removal of radioactive cesium in water. J. Mater. Chem. A.

[62] Ihsanullah (2019). Carbon nanotube membranes for water purification: Developments, challenges, and prospects for the future. Sep. Purif. Technol..

[63] Yan H., Wu H., Li K., Wang Y., Tao X., Yang H., Li A., Cheng R. (2015). Influence of the Surface Structure of Graphene Oxide on the Adsorption of Aromatic Organic Compounds from Water. ACS Appl. Mater. Interfaces.

[64] Das R. (2017). Carbon Nanotube in Water Treatment. Nanohybrid Catalyst Based on Carbon Nanotube: A Step-By-Step Guideline from Preparation to Demonstration.

[65] Yu J.-G., Zhao X.-H., Yu L.-Y., Jiao F.-P., Jiang J.-H., Chen X.-Q. (2014). Removal, recovery and enrichment of metals from aqueous solutions using carbon nanotubes. J. Radioanal. Nucl. Chem..

[66] Dwivedi C., Kumar A., Ajish J.K., Singh K.K., Kumar M., Wattal P.K., Bajaj P.N. (2012). Resorcinol-formaldehyde coated XAD resin beads for removal of cesium ions from radioactive waste: Synthesis, sorption and kinetic studies. RSC Adv..

[67] Yavari R., Huang Y.D., Ahmadi S.J. (2011). Adsorption of cesium (I) from aqueous solution using oxidized multiwall carbon nanotubes. J. Radioanal. Nucl. Chem..

[68] Yang S., Shao D., Wang X., Hou G., Nagatsu M., Tan X., Ren X., Yu J. (2015). Design of Chitosan-Grafted Carbon Nanotubes: Evaluation of How the –OH Functional Group Affects Cs^+^ Adsorption. Mar. Drugs.

[69] Yang S., Han C., Wang X., Nagatsu M. (2014). Characteristics of cesium ion sorption from aqueous solution on bentonite- and carbon nanotube-based composites. J. Hazard. Mater..

[70] Kaper H., Nicolle J., Cambedouzou J., Grandjean A. (2014). Multi-method analysis of functionalized single-walled carbon nanotubes for cesium liquid–solid extraction. Mater. Chem. Phys..

[71] Jang J., Miran W., Lee D.S. (2018). Amino-functionalized multi-walled carbon nanotubes for removal of cesium from aqueous solution. J. Radioanal. Nucl. Chem..

[72] Vipin A.K., Ling S., Fugetsu B. (2014). Sodium cobalt hexacyanoferrate encapsulated in alginate vesicle with CNT for both cesium and strontium removal. Carbohydr. Polym.

[73] Li T., He F., Dai Y.J. (2016). Prussian blue analog caged in chitosan surface-decorated carbon nanotubes for removal cesium and strontium. J. Radioanal. Nucl. Chem..

[74] Draouil H., Alvarez L., Causse J., Flaud V., Zaibi M.A., Bantignies J.L., Oueslati M., Cambedouzou J. (2017). Copper hexacyanoferrate functionalized single-walled carbon nanotubes for selective cesium extraction. New J. Chem..

[75] Lee H.-K., Choi J.W., Oh W., Choi S.-J. (2016). Sorption of cesium ions from aqueous solutions by multi-walled carbon nanotubes functionalized with copper ferrocyanide. J. Radioanal. Nucl. Chem..

[76] Tsuruoka S., Fugetsu B., Khoerunnisa F., Minami D., Takeuchi K., Fujishige M., Hayashi T., Kim Y.A., Park K.C., Asai M. (2013). Intensive synergetic Cs adsorbent incorporated with polymer spongiform for scalable purification without post filtration. Mater. Express.

[77] Zheng Y., Qiao J., Yuan J., Shen J., Wang A.-J., Niu L. (2017). Electrochemical Removal of Radioactive Cesium from Nuclear Waste Using the Dendritic Copper Hexacyanoferrate/Carbon Nanotube Hybrids. Electrochim. Acta.

[78] Chen F.-P., Jin G.-P., Peng S.-Y., Liu X.-D., Tian J.-J. (2016). Recovery of cesium from residual salt lake brine in Qarham playa of Qaidam Basin with prussian blue functionalized graphene/carbon fibers composite. Colloids Surf. A Physicochem. Eng. Asp..

[79] Dichiara A.B., Sherwood T.J., Benton-Smith J., Wilson J.C., Weinstein S.J., Rogers R.E. (2014). Free-standing carbon nanotube/graphene hybrid papers as next generation adsorbents. Nanoscale.

[80] Sui Z., Meng Q., Zhang X., Ma R., Cao B. (2012). Green synthesis of carbon nanotube–graphene hybrid aerogels and their use as versatile agents for water purification. J. Mater. Chem..

[81] Yang H.-M., Hwang J.R., Lee D.Y., Kim K.B., Park C.W., Kim H.R., Lee K.-W. (2018). Eco-friendly one-pot synthesis of Prussian blue-embedded magnetic hydrogel beads for the removal of cesium from water. Sci. Rep..

[82] Yang H., Li H., Zhai J., Sun L., Zhao Y., Yu H. (2014). Magnetic prussian blue/graphene oxide nanocomposites caged in calcium alginate microbeads for elimination of cesium ions from water and soil. Chem. Eng. J..

[83] Lin Y., Cui X. (2005). Novel hybrid materials with high stability for electrically switched ion exchange: Carbon nanotube–polyaniline–nickel hexacyanoferrate nanocomposites. Chem. Commun..

[84] Gautam R.K., Chattopadhyaya M.C., Gautam R.K., Chattopadhyaya M.C. (2016). Chapter 13 - Nanomaterials in the Environment: Sources, Fate, Transport, and Ecotoxicology. Nanomaterials for Wastewater Remediation.

[85] Liu X.T., Mu X.Y., Wu X.L., Meng L.X., Guan W.B., Ma Y.Q., Sun H., Wang C.J., Li X.F. (2014). Toxicity of Multi-Walled Carbon Nanotubes, Graphene Oxide, and Reduced Graphene Oxide to Zebrafish Embryos. Biomed. Environ. Sci..

[86] Qi W., Tian L., An W., Wu Q., Liu J., Jiang C., Yang J., Tang B., Zhang Y., Xie K. (2017). Curing the Toxicity of Multi-Walled Carbon Nanotubes through Native Small-molecule Drugs. Sci. Rep..

[87] Das R., Abd Hamid S.B., Ali M.E., Ismail A.F., Annuar M.S.M., Ramakrishna S. (2014). Multifunctional carbon nanotubes in water treatment: The present, past and future. Desalination.

[88] Fujikawa Y., Ozaki H., Tsuno H., Wei P., Fujinaga A., Takanami R., Taniguchi S., Kimura S., Giri R.R., Lewtas P., Nakajima K. (2015). Volume Reduction of Municipal Solid Wastes Contaminated with Radioactive Cesium by Ferrocyanide Coprecipitation Technique. Nuclear Back-End and Transmutation Technology for Waste Disposal: Beyond the Fukushima Accident.

[89] Bartoś B., Filipowicz B., Łyczko M., Bilewicz A.J. (2014). Adsorption of ^137^Cs on titanium ferrocyanide and transformation of the sorbent to lithium titanate: A new method for long term immobilization of ^137^Cs. J. Radioanal. Nucl. Chem..

[90] Doumic L.I., Salierno G., Ramos C., Haure P.M., Cassanello M.C., Ayude M.A. (2016). “Soluble” vs. “insoluble” Prussian blue based catalysts: Influence on Fenton-type treatment. RSC Adv..

[91] Kim Y., Kim Y.K., Kim S., Harbottle D., Lee J.W. (2017). Nanostructured potassium copper hexacyanoferrate-cellulose hydrogel for selective and rapid cesium adsorption. Chem. Eng. J..

[92] Shih C.-J., Lin S., Sharma R., Strano M.S., Blankschtein D. (2012). Understanding the pH-Dependent Behavior of Graphene Oxide Aqueous Solutions: A Comparative Experimental and Molecular Dynamics Simulation Study. Langmuir.

[93] Kashyap S., Mishra S., Behera S.K. (2014). Aqueous Colloidal Stability of Graphene Oxide and Chemically Converted Graphene. J. Nanopart..

[94] Namvari M., Namazi H. (2014). Clicking graphene oxide and Fe_3_O_4_ nanoparticles together: An efficient adsorbent to remove dyes from aqueous solutions. Int. J. Environ. Sci. Technol..

[95] Tsukada T., Uozumi K., Hijikata T., Koyama T., Ishikawa K., Ono S., Suzuki S., Denton M.S., Keenan R., Bonhomme G. (2014). Early construction and operation of highly contaminated water treatment system in Fukushima Daiichi Nuclear Power Station (I)—Ion exchange properties of KURION herschelite in simulating contaminated water. J. Nucl. Sci. Technol..

[96] Prevost T., Blase M., Paillard H., Mizuno H. (2012). Areva’s Actiflo trademark -Rad water treatment system for the Fukushima nuclear power plant. ATW Int. Z. Fuer Kernenerg..

[97] Lehto J., Koivula R., Leinonen H., Tusa E., Harjula R. (2019). Removal of Radionuclides from Fukushima Daiichi Waste Effluents. Sep. Purif. Rev..

[98] Saini A., Koyama T. (2016). Cleanup technologies following Fukushima. MRS Bull..

[99] Dichiara A.B., Weinstein S.J., Rogers R.E. (2015). On the Choice of Batch or Fixed Bed Adsorption Processes for Wastewater Treatment. Ind. Eng. Chem. Res..

[100] Chen G.-R., Chang Y.-R., Liu X., Kawamoto T., Tanaka H., Kitajima A., Parajuli D., Takasaki M., Yoshino K., Chen M.-L. (2015). Prussian blue (PB) granules for cesium (Cs) removal from drinking water. Sep. Purif. Technol..

[101] Ikarashi Y., Mimura H., Nakai T., Niibori Y., Ishizaki E., Matsukura M. (2014). Selective Cesium Uptake Behavior of Insoluble Ferrocyanide Loaded Zeolites and Development of Stable Solidification Method. J. Ion Exchange.

[102] Gu B.X., Wang L.M., Ewing R.C. (2000). The effect of amorphization on the Cs ion exchange and retention capacity of zeolite-NaY. J. Nucl. Mater..

[103] Abusafa A., Yücel H. (2002). Removal of ^137^Cs from aqueous solutions using different cationic forms of a natural zeolite: Clinoptilolite. Sep. Purif. Technol..

[104] Nakamoto K., Ohshiro M., Kobayashi T. (2017). Mordenite zeolite—Polyethersulfone composite fibers developed for decontamination of heavy metal ions. J. Environ. Chem. Eng..

[105] Nakajima L., Yusof N.N.M., Kobayashi T. (2015). Calixarene-Composited Host–Guest Membranes Applied for Heavy Metal Ion Adsorbents. Arab. J. Sci. Eng..

